# Gene-Disease Interaction Retrieval from Multiple Sources: A Network Based Method

**DOI:** 10.1155/2016/3594517

**Published:** 2016-07-13

**Authors:** Lan Huang, Ye Wang, Yan Wang, Tian Bai

**Affiliations:** ^1^College of Computer Science and Technology, Jilin University, Changchun 130012, China; ^2^Key Laboratory of Symbolic Computation and Knowledge Engineering, Jilin University, Ministry of Education, Changchun 130012, China

## Abstract

The number of gene-related databases has been growing largely along with the research on genes of bioinformatics. Those databases are filled with various gene functions, pathways, interactions, and so forth, while much biomedical knowledge about human diseases is stored as text in all kinds of literatures. Researchers have developed many methods to extract structured biomedical knowledge. Some study and improve text mining algorithms to achieve efficiency in order to cover as many data sources as possible, while some build open source database to accept individual submissions in order to achieve accuracy. This paper combines both efforts and biomedical ontologies to build an interaction network of multiple biomedical ontologies, which guarantees its robustness as well as its wide coverage of biomedical publications. Upon the network, we accomplish an algorithm which discovers paths between concept pairs and shows potential relations.

## 1. Introduction

Gene data of many kinds has been increasing sharply since the gene sequencing technology has been developed and improved over decades. Each gene database is built to store and manage a certain kind of gene features. When researchers need to get multiple features of some genes, they need to go through many gene databases, which is quite inefficient [1–5]. Besides, those gene databases usually cover genes of many species, from bacteria to animals [6]. Some gene features extracted from those databases are not related to human or human diseases; thus, there are a lot of noises from gene databases while extracting gene features for biomedical research [7]. As in this paper, we intend to implement a human gene relation network based on similarity with extension of human diseases for biomedical researchers. And as it is pointed out above, there are mainly two obstacles to achieve our goal.

- The distributed storage and management of gene features in various databases make it inefficient to gather all gene-related features. Researchers need to query many gene databases to get enough information on required genes.
- Most of gene databases contain multiple gene information from various species. Most commonly, lots of gene databases include gene information from yeast and mice. Some of the gene information from this kind of databases is difficult in building connections to biological process of human or human diseases. Such kind of nonrelated information may be confusing for biomedical researchers.

In order to implement a gene-related network, we gather several gene features from gene databases. Gene symbols are widely acknowledged and used in most literatures on biomedical research to indicate genes. We adopt gene symbols as fundamental frame for gene relation network, for it is extensively used and is robust. Upon this frame, all collected gene features are put in vectors and are calculated by similarity algorithm, which is defined in next chapter, to generate a similarity matrix of genes. The prototype of gene relation network is extracted with a threshold value from the matrix.

There are many gene-disease relations that have been verified in biomedical literatures [14–22]. Those literatures have been digitized over years and researchers have developed ways to extract relation pairs, such as gene relation pairs, from the literatures. The approaches that have been developed so far can be divided into two categories in general: cooccurrence algorithms and expert inspection. Those two categories of approaches to extract relations from literatures both have advantages and drawbacks.

- Cooccurrence algorithms are relatively high in efficiency and can process the literature databases like Medline with no or little supervision from researchers. Therefore, this category of approaches can process a lot of literatures and newly published articles to get massive and up-to-date results. Besides, it can also improve itself by adopting latest cooccurrence algorithms.
- Expert inspection, on the other hand, surpasses cooccurrence algorithms in accuracy. Since relation pairs in biomedical demand high precision, it is hard to replace expert inspection with cooccurrence algorithms now. Expert inspection is realized by a group of specialists or a domanial community. It is considerably low in efficiency, coverage of literatures, and self-improvement. It takes time for a group of specialists or a domanial community to accept new knowledge, so the relation pairs extracted this way are more conservative.

In order to extend the gene relation network to human diseases and not to obtain the drawbacks of either category of relation pair extraction approaches, we adopt a two-step extension method. In the first step, we extend gene relation network to human diseases with databases created through expert inspection approach, in order to ensure the accuracy of gene-disease relations. Second step, we map disease ontology to the disease names of the databases that are used. The second step makes it possible to append relations through cooccurrence approaches or to implement gene-disease relation retrieval.

So we implement a broader retrieval of human gene-disease relations by building an extended human gene-disease network, which is realized by combining gene relations, disease ontology, and genetic association database. Any interaction of genes and diseases within limited paths may indicate an uncovered relation in biomedical research and the network can be a useful method to instruct relation discovery of human gene-disease.

**Figure 1:**
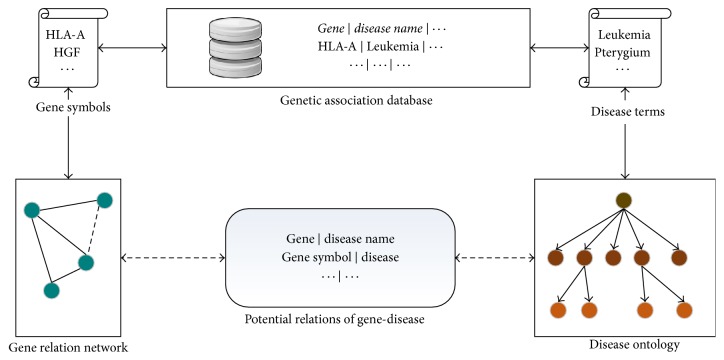
Extended gene-disease network.

The network in Figure 1 is the main method to overcome the drawbacks of current databases. Genetic association database (GAD) provides solid relations between human gene and disease, while gene relation network and disease ontology function as extensions to implement broader coverage of genes and diseases. Those pairs of gene and disease that cannot be found in GAD may possibly be discovered in extended gene-disease network. The pairs that are found in our network rather than GAD may be potential relations of gene-disease.

In this paper, we first build gene relation network and combine GAD with gene relation network and disease ontology to form extended gene-disease network. Then we apply an extended retrieval algorithm on this network and verify the validity of this method.

## 2. Materials and Methods

### 2.1. Gene Databases, Disease, and Gene-Disease Relation Database

The fact that one gene relates to another gene or that two genes are similar means either that one gene interacts with another gene or that both genes share the same pathway, gene function, biological process, and so forth. So multiple gene databases that contain such knowledge are needed to build gene similarity. We collect those features from dbSNP [23], DrugBank [24], Ensembl [25], KEGG [26], GenBank [27], NCIPID [28], Reactome [29], STRING [30], UniGene [31], and Gene Ontology [32] as in Table 1. We use bioDBnet to convert all gene-related ID from database above to gene symbol of HUGO gene nomenclature committee.

Gene symbols are introduced as frame to present genes and to manage gene relations. Since gene symbols are commonly used in biomedical literatures and databases, there is no need to convert relations from databases mentioned above to gene symbol, which avoids precision loss and ambiguousness. Therefore, the information of those databases can be fully and precisely imported. We adopt the whole set of gene symbol to ensure a wide coverage.

Disease names have several widely used thesauri in biomedical literatures. We adopt disease ontology as the fundamental thesaurus for disease names and MeSH, UMLS, SNOMED-CT, and ICD10 as supplement in Table 2.

Many disease ontology terms have references to the other thesauri, and it is possible to link the other four thesauri to disease ontology. In this way, we extend the coverage of disease names and maintain the tree structure of ontology.

We introduce genetic association database (GAD, Table 3) [33] as gene-disease relation database. The GAD is a database that displays an archive of the results of genetic association studies of complex human diseases and disorders. This database has been retired in 2014, but there are over a hundred thousand lines in it. In this paper, we apply this database to build links between genes and human diseases.

### 2.2. Gene Similarity and Extended Gene Network

Genes interact with each other in many ways, and there are few databases that contain gene interactions of all kinds. We use gene relations to extend existing gene-disease relations, so the interaction types of genes are not important in our network and all types of interactions are introduced to build gene relation network equally.

Before building a gene network, we need to calculate the similarity of genes. First, we extract gene-related information from the 10 databases mentioned above and create gene vector as follows:

 〈Gene  Symbol, (gene  features  list  from  dbSNP),…, (gene  features  list  from  GO)〉.

Each gene has a corresponding gene vector and we need to compare each pair of genes and calculate the similarity value according to the equation below:

(1) Sgs,gt=∑m=1nL1∩L2−L1∪L2/2L1∪L2/2×αm,∑m=1nαm=1,

where *S*(*g*_*s*_, *g*_*t*_) is the similarity value of gene_*s*_ and gene_*t*_, *L* is the list of features from gene relation databases, *α*_*m*_ is the adjustment parameter for each database and the default value for each *α*_*m*_ is 1/*n*, and *n* is the number of databases.

In this way, we get the similarity value of each gene pairs and the value is from −1 to 1. The greater the value is, the more similar the genes are, and, according to the feature we selected, genes with higher similarity values are more likely to appear in the same gene functions, cellular components, and gene pathways. We build up the similarity matrix of genes, where the horizontal axis and vertical axis are both lists of genes; the value in the matrix is the similarity value of the corresponding genes on axes. Consider

(2) $$\begin{array}{c} \text{Sig}\left(\;n\;\right)=\frac{e^{\left|\sum_{m\neq n}S\left(g_{m},g_{n}\right)\right|}}{N-1}\underset{m\neq n}{\sum }\frac{\left|S\left(g_{m},g_{n}\right)\right|}{S\left(g_{m},g_{n}\right)}, \end{array}$$

where Sig(*n*) is the importance of a node and *N* is the number of nodes. Since the similarity value is from −1 to 1, we consider that genes with values below 0 are not similar and those whose values above 0 are similar. We first sum up the sign value of one gene with all other genes to determine the similarity of this gene within all genes. As we consider before, the total sign represents the similarity of this gene. If the absolute value of similarity is close to 1, it means that those genes are either very much alike or are not alike at all. Those values describe the character of gene; we use exponential function to enhance the value. Those gene with a high absolute value has a strong connection within or without all other genes, which means this gene is more significant than others. The greater the value is, the more important the node is in the network. It helps us to find important nodes and to determine a threshold value for the similarity matrix.

Since −1 means that the gene pair has nothing in common, we need to eliminate such gene pairs. The threshold value of similarity is 0 by default and can vary from −1 to 1 to control the credibility of similarity of gene pairs. Those gene pairs with similarity value greater than threshold are activated in the gene relation network.

### 2.3. Broader Human Gene-Disease Relations with Extension of Genes and Diseases

We use genetic association database to build links between genes and diseases. The gene thesaurus of GAD is gene symbol, the same as the thesaurus of gene relation network, so the GAD can be directly linked to the gene relation network. Through the internal links of GAD, gene relation network is extended to gene-disease relation network.

However, the disease names of GAD cannot be linked to disease ontology directly. So, we need to develop method to link GAD to disease ontology.

First, we extract disease names from genetic association database and match them to disease names in disease ontology. As we can see, only about one-third of the disease names can be matched to disease ontology. Since GAD is a relatively small database of disease-gene relation, this matching rate is unacceptable, because too much useful information or knowledge is wasted. Therefore, we introduce several widely used disease thesauruses to improve the mapping result.

Almost every term in disease ontology has one or more x-refs or synonyms. Synonyms barely improve the mapping rate, because the synonyms are too similar to the disease ontology name and not every disease in DO has one. Since all data in GAD is extracted from biomedical literatures and MeSH is a standard thesaurus in this field and is among the x-refs in disease ontology, it is reasonable to introduce MeSH and some other thesauri to extend the vocabulary of disease ontology. Those disease names that cannot be mapped directly to disease ontology now can be matched in MeSH and mapped indirectly to disease ontology through x-refs. In this way, over half of the disease names from GAD can be linked to disease ontology, but the rate is still too low.

In order to find out the reason why so many disease names from GAD cannot be mapped to disease ontology, we manually check the disease names by randomly selecting some of the names and then scanning multiple disease thesauruses for the selected names. Most of the disease names do not come up in those thesauruses. However, some diseases with similar names are in the result list. Such kinds of diseases are not the same by name but are the same type of diseases. So we consider such kind of disease names as the child nodes of a disease with the largest number of similar names. In this way, over 96% of the disease names have at least one link to disease ontology as in Table 4.

The rest of the names that cannot be linked to disease ontology are initials for diseases, medical test, or other disease related factors that are not diseases. We build a chart separately for these names. Examples of those 4 methods are in Table 5.

### 2.4. Gene-Disease Network

As in Table 6, there are 3 kinds of edges, which represent 3 kinds of relations. The gene-gene relations are built based on the similarity of genes. They are calculated within pathways, interaction, and biological process of 10 databases. The disease-disease relations are mainly the* “is-a”* relationship of disease ontology. Other kinds of disease-disease relations are indirect link from GAD diseases to disease ontology terms and disease related terms that are not diseases, such as symptoms. The gene-disease relations are all internal links of GAD and are all reliable.

### 2.5. Disease Name Similarity Score Function

Since a great number of diseases from various sources cannot be linked to disease ontology terms directly, we adopt ambiguous method and x-ref method to map more disease names to disease ontology terms at the expanse of accuracy of the linkage. As is given in Table 5, “Testicular Neoplasms” are related to “Testicular Disease” but they are not equal. Therefore, a similarity score function is developed for disease names to give a computable confidence of two disease names.

We take “Diabetes, Type 2” and “Type 2 Diabetes Mellitus”, “Testicular Neoplasms”, and “Testicular Disease” as examples to demonstrate how the function scores.

First, disease names are divided into individual words. If the disease names have only one word, ignore this step. If there is a comma in the name, put the words after the comma in front of the word before comma. For example, “Diabetes, Type 2” is divided into three words: “Diabetes”, “Type”, and “2”. Then switch “Diabetes” and “Type” and “2”. Finally, we get a list of words: “Type”, “2”, and “Diabetes”.

Second, each word in the word list gets an initial value, and the total value of the words in a list equals 1. After observing some disease names, we consider the word at the end of the list to be more important than the word at the top of the list, for “Diabetes” is more meaningful than “Type”. Then, if there is only one word in the list, its initial value equals 1. Otherwise, the initial value of the former word equals half of the initial value of the latter word in the list, unless the former word is the first word in the list. In this case, the initial value of the first word in the list equals the second. For word list “Type”, “2”, “Diabetes”, and “Mellitus”, the initial values for each word are 0.125, 0.125, 0.25, and 0.5, respectively.

Below is the similarity score function:

(3) $$\begin{array}{c} \text{SC}\left(\;{\text{dis}}_{m},{\text{dis}}_{n}\;\right)=\overset{\text{length}\left(m\right)}{\underset{s=1}{\sum }}\text{IV}\left(\;s\;\right)\varphi \times \overset{\text{length}\left(n\right)}{\underset{t=1}{\sum }}\text{IV}\left(\;t\;\right)\omega , \end{array}$$

where SC(dis_*m*_, dis_*n*_) is the score of disease names *m* and *n*, *φ* and *ω* are valid value for *m* and *n*, and IV is the initial value. If a word in list of disease *m* is found in list of disease *n*, then its *φ* equals 1, otherwise it equals 0. Similarly, if a word in list of disease *n* is found in list of disease *m*, then its *ω* equals 1, otherwise it equals 0.

For disease names mapped to disease ontology directly or through x-ref, they have a score of 1. For disease names mapped with ambiguous method, they get value from similarity function. For example, “Diabetes, Type 2” and “Type 2 Diabetes Mellitus” have a similarity score of 0.5, while “Testicular Neoplasms” and “Testicular Disease” have a similarity score of 0.25.

### 2.6. Confidence Function for Disease-Gene Interactions

Since we have defined similarity score for disease-disease and gene-gene, each disease-gene interactions now can be valued.

The confidence function is

(4) $$\begin{array}{c} \text{DGI}\left(\;d,g\;\right)=\underset{s\in D}{\prod }\text{SC}\left(\;d,s\;\right)\underset{t\in G}{\prod }S\left(\;g,t\;\right), \end{array}$$

where DGI(*d*, *g*) is confidence value of disease *d* and gene *g*. *D* is the set of all diseases, and *G* is the set of all genes.

If there is a path between disease *d* and gene *g*, then DGI(*d*, *g*) ≠ 0 should always be true. However, *S*(*g*, *t*) can be zero even there is a path, so the value of gene similarity needs to be optimized. A path means a connection between two terms either directly or indirectly.

The optimized function is

(5) DGI′d,g=SC′d,sS′g,t,

(6) SC′d=10∏s∈DSCd,s,

(7) S′g=10∏t∈GSg,t+1/2.

DGI′(*d*, *g*) is over 0 as long as there is a path between gene and disease. The value of DGI′(*d*, *g*) ⊂ (0,100), where 100 means that the interaction exists and has been verified. This is because when (5) equals 100, it means that the exponents of (6) and (7) are both 1, which means by definition that there are direct connections.

### 2.7. Expanded Retrieval of Human Genes and Diseases

There are over 100,000 edges in the gene-disease relation network. Each pair of gene and disease is theoretically connected. We develop an algorithm to get pathways between gene and disease under certain conditions.

We consider (term 1, term 2) as a set of two terms, and 〈term  1, term  2〉 denotes that they are connected by an edge. Each term has a value that equals the number of edges that connected to it. The input term pair from users should be disease and gene, and the term pairs generated in the algorithm can be diseases, genes, or disease and gene. Each term is a node in the network. Consider

(8) $$\begin{array}{c} W\left(\;n\;\right)=e^{\left(E_{o}^{n}-E_{i}^{n}\right)}\times E_{o}^{n}\times \varepsilon . \end{array}$$

*W*(*n*) is the score for each node. *E* is the edges of a term, and those edges that connect to input terms are in-edges (*E*_*i*_), and others are out-edges (*E*_*o*_). *ε* is a random number from 0.5 to 1 to provide flexibility, and if this node is already in the list, it equals zero. This equation provides the heuristic function of the following algorithm.

**Figure 2:**
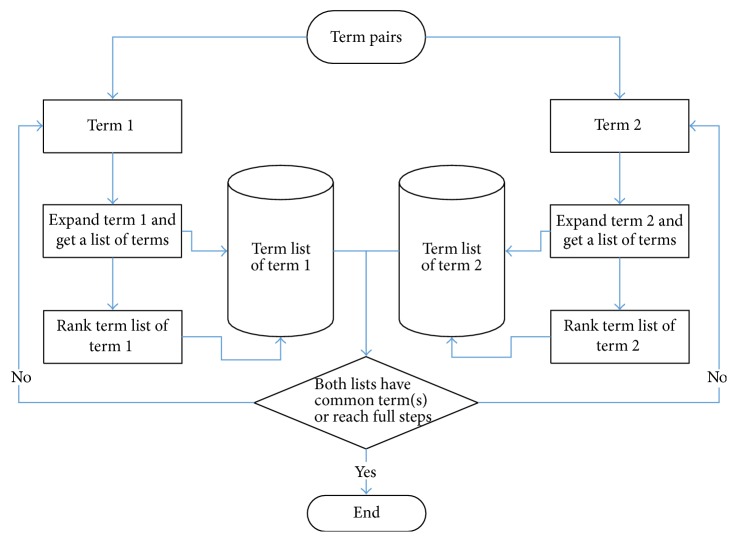
Workflow of algorithm.

Path detection algorithm: first, we input a pair of terms (term 1, term 2) and maximum length of the path (maxlength). Then we search from both terms and record the path length of the expended layers. When the sum of both path length exceed maxlength, the algorithm stops. Otherwise, for one term, all of its neighbors are found and sorted by their value and then added to the end of the list of this term. If there is a duplicated term in the list, change the value of the node to zero. If the intersection of lists of both terms is empty, get the first nonzero term from both lists, delete the headmost terms and extend them to their neighbors, and check again. If the intersection is not empty, return the shared term and its path towards the input terms. The procedures of the algorithm are in Figure 2.

## 3. Experiment and Results

### 3.1. Experiment Design

**Figure 3:**
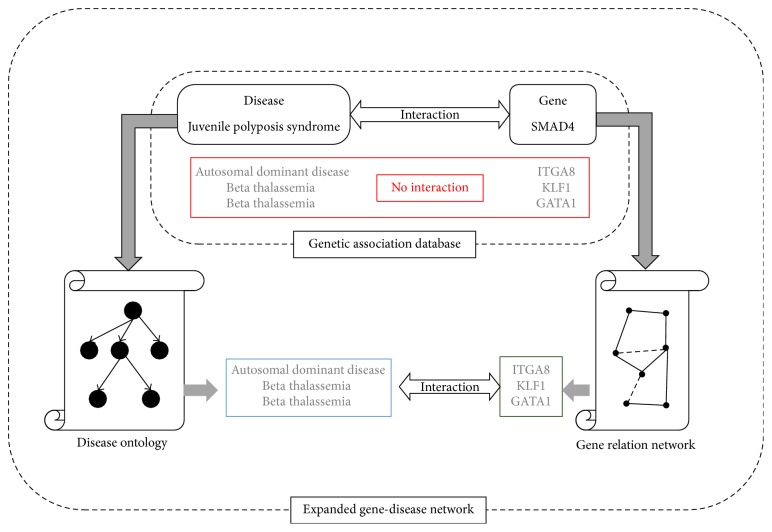
New paths for GAD pairs in expanded gene-disease network [34–36].

The experiment is designed as in Figure 3. We collect PubMed articles and extract gene-disease pairs. Since GAD ceases to update in 2014, those relation pairs extracted from PubMed articles in 2015 are very likely not in our gene-disease network. We apply our path detection algorithm to find paths of gene-disease pairs from PubMed articles in 2015 and test how many of them can be found in our network.

We collect all articles from October, 2015, to November, 2015, in PubMed and extract gene-disease pairs from these articles. We obtain 172686 pieces of articles and extract 159259 pairs of gene-disease. We then apply our algorithm on both GAD and our expanded gene-disease network. The results can be divided into three categories, as shown in Table 7.

### 3.2. Results Analysis

The results can be put in 3 categories. The first kind of gene-disease pairs can be found directly in GAD. The second kind cannot be found directly in GAD, but there are paths to connect them in our network. The third kind can be found directly in GAD, and there are other paths to connect them in our network.

There is a small number of gene-disease pairs that can be found in GAD directly. Since there is no relation attribute of edges in our network or GAD, these gene-disease pairs can represent either new relations or existing ones. The reason of the low percentage is that those gene-disease pairs are extracted from most recent literatures in PubMed while the GAD has ceased to update. It is reasonable that GAD cannot cover most new pairs. Most of these pairs are extracted from literatures which explain new relations or make comparisons.

From Table 7, we can see that over 4/5 pairs of gene-disease can be found in our network alone. The expanded networks of genes and diseases contribute the most in discovering this high percentage of gene-disease pairs. Disease ontology terms and other reference medical thesauri unify disease names and allow ambiguous match of disease names. Gene relation network provides similarities between genes. Its connectivity is controlled by the threshold value of similarity. In this experiment, we set an intermediate threshold value for gene relation network and allow ambiguous match of disease name. So the result is relatively high in number, and the relations between the gene-disease pairs are relatively weak. It is possible to control the parameters of the expanded gene-disease network and get a looser or tighter result. The parameters can help the network to fit in different needs of retrieval.

There are new paths for gene-disease pairs in expanded network that can be found in GAD. From Table 8, many gene-disease pairs do not have alternative paths, because many diseases and related genes have already formed a small group in the network, and they have reached their high connectivity. However, those that have new paths are more intriguing, because they may potentially represent new relations that have yet been proved. This indicates that even though GAD has no relations of those pairs, our expanded network still can give paths to connect them. Tables 9 and 10 are examples of intermediate steps of the experiment.

Poon et al. [37] developed a method to extract medical related knowledge from PubMed and demonstrate it on web. It accomplished a simple reasoning function to show potential term pairs that may interact through a third term. There are resemblances between their work and ours, but those two works have different goals and different method to fulfill it. Comparisons are in Table 11.

## 4. Conclusion

We implement a gene-disease relation network based on gene symbols and disease ontology. The network contains three kinds of relations, and all terms are linked to others within unlimited length of path. By controlling the length path between two terms, we can discover reasonable pathway between terms which have never been brought up together. We test latest research outcome in our network and some of them are found in the network even before the articles. So the potential relations in the network can be used to inspire other researchers.

## 5. Future Work

We accomplished score algorithms and retrieval algorithm in this paper on sample set of genes and diseases from multiple sources. In future, we will apply our methods on complete sources of genes and diseases and import other medical related terms like phenotype and provide a fully functional platform as service for users. Meanwhile, the retrieval algorithm will be improved to ensure efficiency on huge data.

## Figures and Tables

**Table 1:** Gene relation databases.

Database name	Relation type	URL
dbSNP	ID conversion	http://www.ncbi.nlm.nih.gov/SNP/
DrugBank	ID conversion	http://www.drugbank.ca/
Ensembl	ID conversion	http://asia.ensembl.org/index.html?redirect=no
KEGG	Pathway	http://www.genome.jp/kegg/
GenBank	Gene-protein	http://www.ncbi.nlm.nih.gov/genbank/
NCIPID	Pathway	https://pid.nci.nih.gov/
Reactome	Pathway	http://www.reactome.org/
STRING	Interaction	http://string-db.org/
UniGene	ID conversion	http://www.ncbi.nlm.nih.gov/unigene/
GO	ID conversion	http://geneontology.org/

**Table 2:** Disease name thesauri.

Thesaurus name	URL
Disease ontology [8]	http://disease-ontology.org/
MeSH [9]	https://www.nlm.nih.gov/mesh/
UMLS [10]	https://www.nlm.nih.gov/research/umls/
SNOMED-CT [11]	https://www.nlm.nih.gov/research/umls/ Snomed/snomed_main.html
ICD10 [12]	http://www.who.int/classifications/icd/en/

**Table 3:** Genetic association database (part).

Disease	Dis_class	Gene
Leukemia	CANCER	HLA-A
Alzheimer's disease	NEUROLOGICAL	HFE
Thalassemia	HEMATOLOGICAL	HBA1
Emphysema	CARDIOVASCULAR	GSTT1
PAH metabolites, urinary	METABOLIC	GSTT1

**Table 4:** Percentage of matched disease names.

Method	Direct match	Half ambiguous	With x-ref	Ambiguous
Percentage	10%	45%	65%	96%

**Table 5:** Examples of four methods.

	Disease name from GAD	Disease name from thesaurus
Direct match	Leukemia	Leukemia
Half ambiguous	Diabetes, Type 2	Type 2 Diabetes Mellitus
With x-refs	Sleep Disorders	Sleep Disorder
Ambiguous	Testicular Neoplasms	Testicular Disease

**Table 6:** Nodes and edges of the network.

Edge	Number of edges	Number of nodes
Gene-gene	207051075	18998
Disease-disease	6932	6588
Gene-disease	121309	25586

**Table 7:** Distribution of test results.

Results	In GAD	Only in expanded network	All
Percentage of total	3.2%	82.2%	85.4%
Count	5120	130918	159259

**Table 8:** Alternative paths comparison of pairs in GAD.

Results	Without alternative paths	With alternative paths
Percentage	71.5%	28.4%
Count	3663	1457

**Table 9:** Results of alpha thalassemia and beta thalassemia in GAD.

Disease	Related genes
Alpha thalassemia	Hb, HP, UGT1A1, and ABO
Beta thalassemia	HBG2, PROCR, NOS1, NOS2, HBG1, F2, F5, COL1A1, HAMP, VDR, TNF, SERPINE1, AHSP, ITGB3, APOE, APOB, LARGE, HBBP1, HLA-C, GSTT1, GSTM1, FGB, F13A1, ESR1, ACE, and COL1A1
Alpha and beta thalassemia	MYB, MTHFR, HBA@, HBS1L, HBB, HBA2, HBA1, G6PD, HBB, BCL11A, UGT1A1, and HFE

**Table 10:** Examples of gene-disease pairs found in GAD.

Gene	Disease
THBS1	Prostate cancer
TH	Mental disorder
TH	Mood disorder
TH	Borderline personality disorder
MB	Breast cancer
TNF	Leptospirosis
PC	Colorectal cancer

**Table 11:** Comparisons of Literome, DisGeNet, and expanded gene-disease network.

	Literome	DisGeNet [13]	Expanded gene-disease network
Sources	PubMed	GAD, CTD, and other 12 more	GAD, PubMed, and 14 more databases
PubMed articles linkage	Yes	No	Partial
Gene-disease interactions	Yes	Yes	Yes
Interaction path	Partial	No	Yes
Path length	3	1	Can be assigned
Demonstration	PMID and marked texts	List of triplet	Disease names and gene names in path
Database update	Yes	Yes	Yes
Supported medical terms retrieval	Genic interactions, genotype-phenotype	Disease and gene	Gene and disease interactions
